# A BCR-ABL Mutant Lacking Direct Binding Sites for the GRB2, CBL and CRKL Adapter Proteins Fails to Induce Leukemia in Mice

**DOI:** 10.1371/journal.pone.0007439

**Published:** 2009-10-13

**Authors:** Kara J. Johnson, Ian J. Griswold, Thomas O'Hare, Amie S. Corbin, Marc Loriaux, Michael W. Deininger, Brian J. Druker

**Affiliations:** 1 Division of Hematology and Medical Oncology, Oregon Health & Science University Knight Cancer Institute, Portland, Oregon, United States of America; 2 Department of Pathology, Oregon Health & Science University, Portland, Oregon, United States of America; 3 Howard Hughes Medical Institute, Oregon Health & Science University Knight Cancer Institute, Portland, Oregon, United States of America; University of Kansas Medical Center, United States of America

## Abstract

The BCR-ABL tyrosine kinase is the defining feature of chronic myeloid leukemia (CML) and its kinase activity is required for induction of this disease. Current thinking holds that BCR-ABL forms a multi-protein complex that incorporates several substrates and adaptor proteins and is stabilized by multiple direct and indirect interactions. Signaling output from this highly redundant network leads to cellular transformation. Proteins known to be associated with BCR-ABL in this complex include: GRB2, c-CBL, p62^DOK^, and CRKL. These proteins in turn, link BCR-ABL to various signaling pathways indicated in cellular transformation. In this study we show that a triple mutant of BCR-ABL with mutations of the direct binding sites for GRB2, CBL, p62^DOK^ and CRKL, is defective for transformation of primary hematopoietic cells *in vitro* and in a murine CML model, while it retains the capacity to induce IL-3 independence in 32D cells. Compared to BCR-ABL, the triple mutant's ability to activate the MAP kinase and PI3-kinase pathways is severely compromised, while STAT5 phosphorylation is maintained, suggesting that the former are crucial for the transformation of primary cells, but dispensable for transformation of factor dependent cell lines. Our data suggest that inhibition of BCR-ABL-induced leukemia by disrupting protein interactions could be possible, but would require blocking of multiple sites.

## Introduction

The BCR-ABL tyrosine kinase is the molecular hallmark of chronic myeloid leukemia (CML) and its kinase activity is required for disease induction [1], [2]. BCR-ABL transforms Rat-1fibroblasts [3] and B-cell precursors [4] *in vitro* and confers interleukin-3 (IL-3) independent growth when expressed in IL-3 dependent myeloid cell lines [5]. In murine bone marrow transplantation/transduction experiments, BCR-ABL infected bone marrow transplanted into mice induces a myeloproliferative syndrome that is transplantable into secondary recipients [6], [7], [8]. Since the tyrosine kinase activity of BCR-ABL is essential for its oncogenic activity *in vitro* and *in vivo* [1], [2], much effort has been directed at determining which of its substrates are required for leukemogenesis. A number of BCR-ABL substrates have been identified, including BCR-ABL itself, CBL, CRKL, the p85 kDa regulatory subunit of phosphoinositide (PI) 3-kinase, p62^DOK^, RAS-GAP, paxillin, and SHC [9]. Co-immunoprecipitation experiments have shown that BCR-ABL forms stable complexes with several of these substrates including CRKL, SHC, CBL, p62^DOK^, and PI3-kinase [9], [10], [11], [12]. In addition, tyrosine phosphorylation of BCR-ABL at specific residues regulates the binding of proteins such as GRB2 [13]. As a result of these interactions many intracellular signaling pathways are activated, including the RAS, AKT and STAT pathways [9], [10], [11], [12]. In the complicated network of interactions that results, the role and relative importance of individual components has been difficult to establish.

To determine the necessity of various proteins for BCR-ABL function, a common approach has been to identify a binding site for a specific protein on BCR-ABL, mutate the site and analyze the effect on BCR-ABL function. The ability of BCR-ABL constructs to transform IL-3 dependent hematopoietic cell lines to factor independent growth is a common tool used to assess BCR-ABL function. For example, tyrosine 177 of BCR-ABL is the binding site for the adaptor protein GRB2, which links BCR-ABL to the RAS pathway [13], [14]. BCR-ABL containing a mutation of this tyrosine to phenylalanine (Y177F) is still able to transform myeloid cell lines to IL-3 independent growth [15]. This Y177F mutant is also capable of inducing leukemia in a murine leukemia model, but the phenotype of the leukemia is lymphoid as opposed to myeloid [16]. Similar results were seen with a mutant lacking the SH2 domain. This BCR-ABL SH2 domain deletion mutant renders myeloid cells lines IL-3 independent [17], [18], [19], and induces a lymphoid leukemia or a CML-like disease in mice, but the disease latency is increased as compared to full length BCR-ABL [20]. The SH2 domain is reported to mediate direct binding of BCR-ABL to CBL [21], [22] and p62^DOK^ [23]. In the C-terminus of BCR-ABL, a proline-rich region is a direct binding site for the adaptor protein CRKL. Deletion mutants in this region are capable of rendering myeloid cells growth factor independent in the background of p210^BCR-ABL^ [24], and are also capable of inducing leukemia in mice in the p185^BCR-ABL^ background [25].

Although mutation of individual domains abolishes the direct interactions of a signaling protein with BCR-ABL, indirect interactions confound the ability to determine the role of a specific protein or pathway in BCR-ABL transformation. For example, direct binding of CRKL to BCR-ABL is abolished in the proline-rich deletion mutant, but CRKL interacts indirectly with BCR-ABL and is still tyrosine phosphorylated [24]. Therefore, to address the role of various signaling pathways simultaneously and to circumvent difficulties posed by the potential for indirect interactions, we created a mutant of BCR-ABL with a tyrosine to phenylalanine mutation at amino acid 177 (Y177F), an SH2 domain deletion (ΔSH2) and a deletion of the C-terminal proline-rich region (ΔPro). We assessed the ability of this triple mutant to transform myeloid cell lines and induce leukemia and analyzed its ability to interact with signaling proteins and activate downstream signaling pathways.

## Results

### The triple mutant of BCR-ABL is capable of inducing growth factor independence

**Figure 1:**
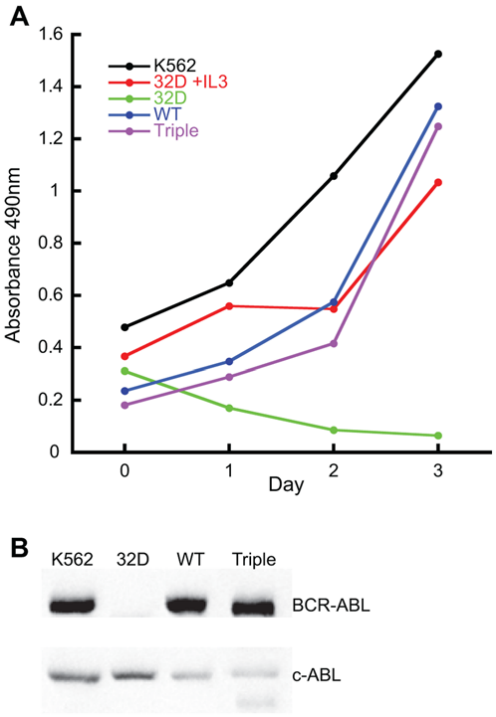
The triple mutant of BCR-ABL is capable of inducing growth factor independence. 32D cell lines matched for BCR-ABL expression were analyzed for their requirement of interleukin-3 (IL-3) for growth. (A) Representative graph of cell proliferation assay on 32D cell lines matched for expression of BCR-ABL grown in the absence of IL-3. (B) Western blot of BCR-ABL (upper panel) and ABL (lower panel) showing relative expression levels of cell lines shown in A.

32D cell lines were generated containing the pSRα vector, full length BCR-ABL without mutations, herein referred to as wild type BCR-ABL (WT), mutants of BCR-ABL containing a tyrosine to phenylalanine mutation at amino acid 177 (Y177F), a deletion of the SH2 domain (ΔSH2), a deletion of a C-terminal proline-rich region (ΔPro) and a triple mutant of BCR-ABL (triple) containing all three. These are reported to be direct binding sites for GRB2 (Y177)[13], [14], CBL[21], [22] and p62^DOK^ (SH2 domain)[23], and CRKL (proline-rich region)[24]. Individual clones were isolated from soft agar cultures and evaluated for expression of BCR-ABL. Several clones expressing BCR-ABL at comparable levels were selected and analyzed for IL-3 dependence by cell proliferation and viability assays. All clones were maintained in the presence of IL-3 prior to assessment of factor independent growth. The parental 32D cells were unable to proliferate in the absence of IL-3, whereas wild type BCR-ABL, the Y177F, ΔSH2, ΔPro and the triple mutant BCR-ABL grew at comparable rates, indicating the triple mutant is capable of inducing factor independent growth. The result of a representative cell proliferation assay with the triple mutant is shown in Figure 1A (data not shown for single mutants). The wild type and triple mutant clones represented in Figure 1 were chosen for further study due to similarity in BCR-ABL expression levels. Growth curve measurements for additional wild type and triple mutant clones (Figure S1) were carried out to confirm that the observed phenotypes were not attributable to random mutational events. Assays of proliferation and viability as determined by trypan blue dye exclusion, performed in the presence and absence of IL-3, yielded similar results, with the triple mutant proliferating and remaining viable at levels comparable to wild type and single mutants in the absence of IL-3 (data not shown). Expression levels of BCR-ABL and a triple mutant clone are shown in Figure 1B.

### Kinase Assays

**Figure 2:**
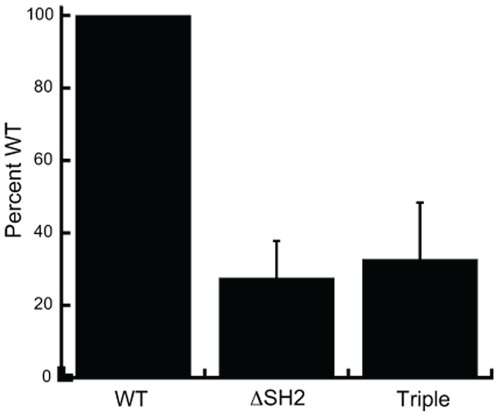
Kinase activity of triple mutant BCR-ABL is 40–50% that of wild type. 32D cells normalized for BCR-ABL expression were immunoprecipitated and kinase activity was assayed using a peptide substrate containing the consensus ABL kinase binding site. Assays were performed in triplicate for each cell line: wild type (WT), ΔSH2 and triple mutant.

It has previously been shown that individual mutations of the Tyr 177 to Phe, and deletion of the SH2 and Proline domains of BCR-ABL do not abolish the associations between BCR-ABL and its substrates [17], [22], [23], [24], [26], nor do they completely abolish the kinase activity [19], [20], [25], [27]. Kinase assays were performed to determine if the kinase activity of BCR-ABL was impaired in the triple mutant. Results, shown in Figure 2, demonstrate the kinase activity of the BCR-ABL triple mutant is 40-50% lower than that of wild type, similar to that of the BCR-ABL mutant lacking only the SH2 domain. The kinase activity of the single mutants has been reported previously by various groups. Cortez et al. investigated the Y177F and SH2 mutants and found they both were able to transphosphorylate an ABL kinase substrate at similar levels to wild type [28]. Ilaria and Roumianstev both saw a decrease in the kinase activity of a ΔSH2 mutant compared to wild type BCR-ABL using phospho-tyrosine immunoblots [19], [20]. Dai [25] and Senechal [27] examined the ΔPro mutation in the p185 and p210 backgrounds, respectively, and also demonstrated that the kinase activity was still present in these mutant forms of BCR-ABL. Since, as reported by these groups, mutation of these domains individually does not affect the interaction of BCR-ABL with its substrates, nor its ability to induce leukemia in mice, we focused our investigation on the triple mutant encompassing each of these component mutants in our studies.

### Protein-protein interactions with BCR-ABL wild type and triple mutant

**Figure 3:**
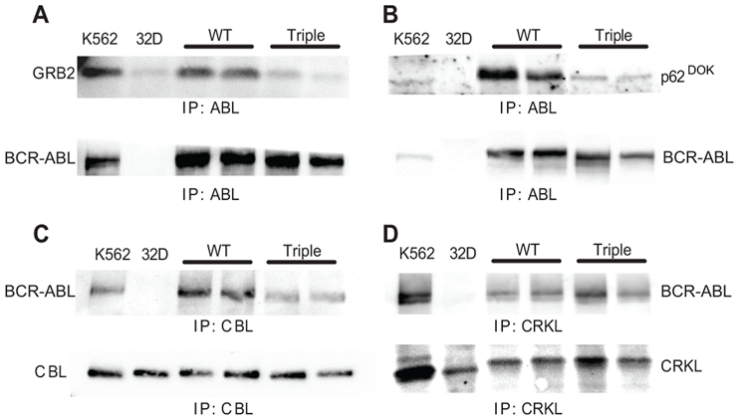
Interaction of BCR-ABL with various adaptor proteins is reduced, but not eliminated in the triple mutant. Lysates from the BCR-ABL expressing 32D cell lines, control line K562 and parental 32D cells were immunoprecipitated (IP) for the indicated proteins and blotted to determine interactions between BCR-ABL and (A) GRB2; (B) p62^DOK^; (C) CBL; and (D) CRKL. Lower panels show normalization for levels of immunoprecipitated proteins: (A and B) BCR-ABL, (C) CBL and (D) CRKL. In (D), the increased amount of BCR-ABL in the triple mutant lane on the left compared to the outermost lane reflects the correspondingly greater amount of immunoprecipitated CRKL in this lane.

To further analyze the biochemical properties of cell lines expressing the triple mutant, whole cell lysates from independent clones were analyzed by immunoprecipitation and immunoblotting. These studies demonstrated that the associations between BCR-ABL and GRB2, p62^DOK^ and c-CBL, were strongly reduced, but not completely eliminated in the triple mutant (Figure 3A, B and C). Surprisingly, the amount of CRKL interacting with the triple mutant remained comparable to that of the wild type clones (Figure 3D), which is similar to previous results with the ΔPro mutant alone [24].

### Tyrosine phosphorylation of BCR-ABL substrates

**Figure 4:**
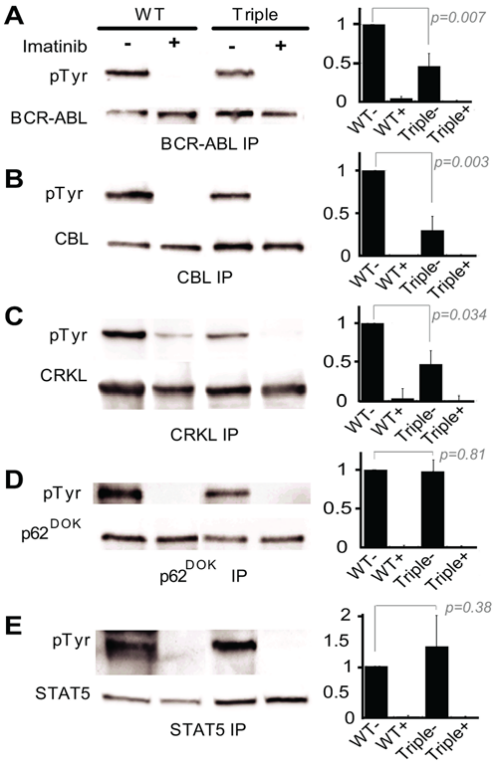
Phosphorylation of various BCR-ABL substrates in wild type and triple mutant BCR-ABL expressing cell lines. Phosphotyrosine levels were determined by immunoprecipitating the indicated proteins from BCR-ABL expressing 32D cell lines and immunoblotting with an anti-phosphotyrosine antibody. The levels of phosphorylation were normalized for the levels of the immunoprecipitated protein, and phosphorylation is reported relative to wild type (right panel). (A) BCR-ABL; (B) CBL; (C) CRKL; (D) p62^DOK^; (E) STAT5. Treatment with 10uM imatinib inhibited phosphorylation of all substrates examined. Data represents three separate experiments, with a representative blot for each shown in the left panels.

The level of tyrosine phosphorylation of BCR-ABL, c-CBL, CRKL, p62^DOK^ and STAT5 were assessed next. Tyrosine phosphorylation levels from three different experiments were quantitated and normalized for total levels of the specific protein being analyzed. The phosphorylation levels relative to wild type (defined as 100%) are shown in Figure 4 along with representative immunoblots. As shown in Figure 4A, the relative level of BCR-ABL tyrosine phosphorylation is reduced by approximately two-fold in the triple mutant as compared to wild type (*p* = 0.007), consistent with the reduced kinase activity. Similarly, CBL, which is a component of the BCR-ABL signaling complex, and CRKL, which is directly associated with and phosphorylated by BCR-ABL, are approximately four-fold (Figure 4B; *p* = 0.003) and two-fold (Figure 4C; *p* = 0.034) less efficiently tyrosine phosphorylated in the context of the triple mutant as compared to wild type BCR-ABL, consistent with reduced BCR-ABL kinase activity and partial disruption of key docking elements within the triple mutant critical to efficient assembly of the BCR-ABL signaling complex. In contrast, the relative tyrosine phosphorylation levels of the downstream BCR-ABL targets p62^DOK^ (Figure 4D; *p* = 0.81) and STAT5 (Figure 4E; *p* = 0.38), are unchanged in the setting of triple mutant BCR-ABL. Wild type BCR-ABL and the triple mutant are equally sensitive to imatinib, as evidenced by complete suppression of tyrosine phosphorylation upon treatment with 10 µM imatinib. In both cases, tyrosine phosphorylation of all examined downstream targets is also abrogated.

### Activation state of signaling pathways

**Figure 5:**
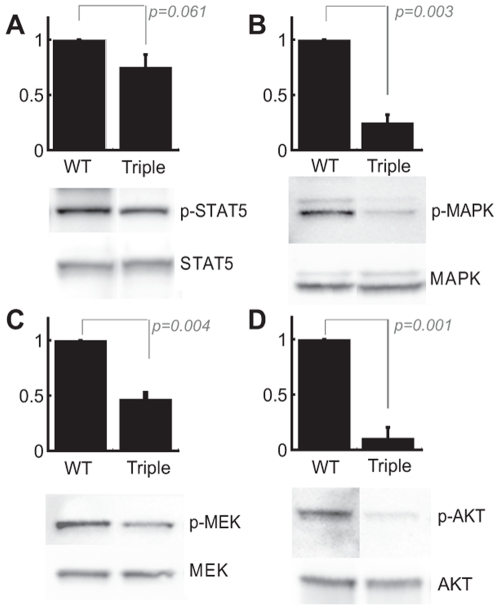
Activation state of various signaling pathways, in the triple mutant BCR-ABL expressing cells, relative to wild type. 32D cell lines expressing either wild type or triple mutant BCR-ABL were lysed and the activation state of the indicated signaling pathways was detected by immunoblotting whole cell lysates with phosphospecific antibodies for each pathway. (A) phospho-STAT5; (B) phospho-MAPK; (C) phospho-MEK; (D) phospho-AKT. The levels of phosphorylation were normalized for the whole cell levels of the protein shown in the lower panel of each immunoblot. (A) STAT5; (B) MAPK; (C) MEK; (D) AKT. Phosphorylation levels are reported relative to wild type (upper panels). Data represents three independent experiments with a representative blot shown.

The activation state of various signaling proteins activated by BCR-ABL (STAT5, MAPK, MEK and AKT) was analyzed in whole cell lysates from cells expressing wild type BCR-ABL and the triple mutant, using phosphospecific antibodies (Figure 5). As with the data presented in Figure 4, the level of phosphorylation from three different experiments was quantitated and normalized for expression of the specific protein being analyzed. The phosphorylation levels relative to wild type (defined as 100%) are shown. The relative level of STAT5 tyrosine phosphorylation was slightly reduced in the triple mutant as compared to wild type, but did not reach statistical significance (Figure 5A; *p* = 0.061), consistent with findings described above (Figure 4E). In contrast, statistically significant reductions in the relative levels of phospho-MAPK (Figure 5B; ∼four-fold; *p* = 0.003), phospho-MEK (Figure 5C; ∼two-fold; *p* = 0.004), and phospho-AKT (Figure 5D; ∼five-fold; *p* = 0.001) were evident in the setting of the BCR-ABL triple mutant.

**Figure 6:**
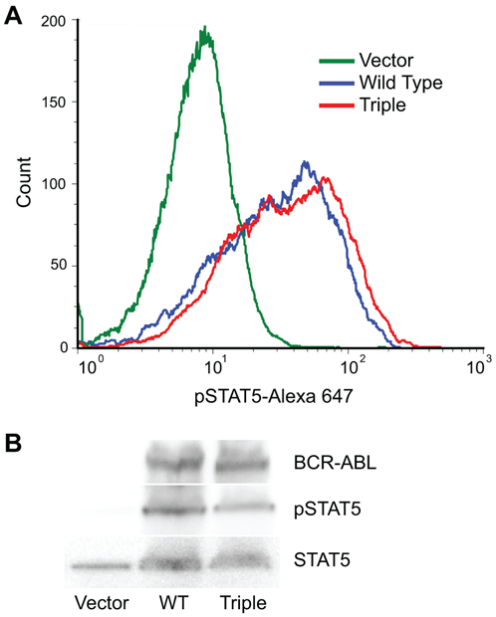
Analysis of phospho-STAT5 in primary cells. GFP positive primary bone marrow transduced with retroviral supernatants from wild type, triple mutant BCR-ABL or vector only was analyzed for the levels of STAT5 phosphorylation by (A) FACS analysis of cells gated for GFP positivity and stained with phospho-STAT5-Alexa 647 antibody or by (B) Western analysis with the indicated antibodies.

The level of STAT5 phosphorylation was also analyzed in primary bone marrow cells infected with matched retroviral stocks for the triple mutant, wild type BCR-ABL or vector control. GFP positive cells were isolated from each of the infected cell groups and the level of STAT5 phosphorylation measured by flow cytometry and western blotting (Figure 6). Similar to the results seen in the cell lines (Figures 4E and 5A), the levels of phospho-STAT5 in bone marrow infected with the triple mutant is comparable to that seen in wild type relative to the vector only control (Figure 6A,B).

### The BCR-ABL triple mutant fails to induce the outgrowth of B-lymphoid cells and myeloid cells

**Figure 7:**
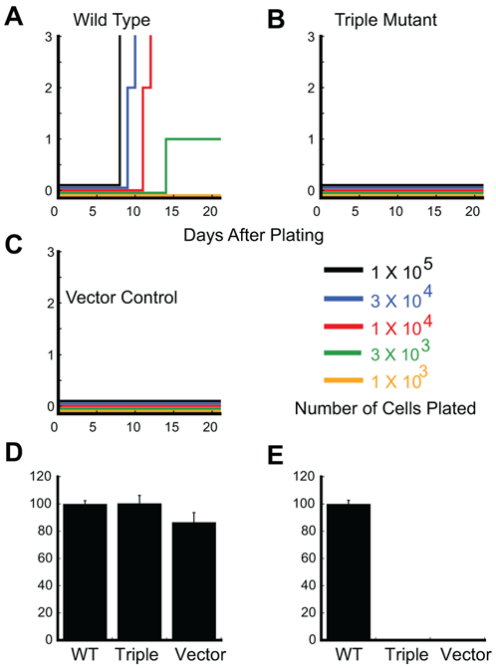
BCR-ABL triple mutant fails to induce the outgrowth of progenitor B-lymphoid cells and myeloid progenitor cells. Transformation of primary B-lymphoid progenitors was assessed in Whitlock/Witte cultures with bone marrow incubated with retroviral supernatants from (A) wild type; (B) triple mutant; and (C) vector only. Cells are plated in serial dilutions from 1×10^5^ (black line), 3×10^4^ (blue line), 1×10^4^ (red line), 3×10^3^ (green line), 1×10^3^ (yellow line), along with 1×10^6^ non-transduced bone marrow cells. Cultures are scored positive for growth when the number of non-adherent cells exceeded 10^6^ per mL of culture medium. Myeloid transformation potential in the presence (D) or absence (E) of cytokines was measured using colony-forming assays of bone marrow transduced with retroviral supernatants from wild type, triple mutant or vector only. were identical in two separate assays.

The ability of the BCR-ABL triple mutant to transform primary B-lymphoid cells was assessed in Whitlock-Witte cultures. In these assays, cells were plated in serial dilutions, from 1×10^3^ cells per well to 1×10^5^ cells per well. The wells were assessed daily for outgrowth, with wells containing at least 1×10^6^ non-adherent cells scored as positive. As shown in Figure 7A, wild type BCR-ABL induced outgrowth at all but the lowest dilution (yellow line). In contrast the triple mutant was unable to induce the outgrowth of B-lymphoid cells at any dilution plated (Figure 7B), similar to the results seen with the MIG vector control (Figure 7C).

The triple mutant was also assessed for transformation ability in myeloid colony forming assays. Normal bone marrow requires the addition of cytokines to support colony formation in semisolid media, while expression of BCR-ABL abrogates this requirement [4]. In methylcellulose cultures of murine bone marrow infected with BCR-ABL and supplemented with IL-3, IL-6 and SCF, there was no statistically significant difference between the ability of the triple mutant and wild type to form colonies (Figure 7D). However in the absence of cytokines, the triple mutant yielded only 4% of the colonies formed in the wild type cultures (Figure 7E). To determine whether the colonies growing under the various conditions contained the BCR-ABL construct, individual colonies were analyzed for GFP by PCR. In the presence of growth factors, 80% of colonies from the vector only and triple mutant cultures expressed GFP compared to 100% of wild type colonies. Though only 4 colonies were present in the triple mutant cultures without growth factors, all expressed GFP (data not shown). Thus, the triple mutant appears to have a profound defect in its ability to transform primary cells as measured in these assays.

### The BCR-ABL triple mutant fails to induce leukemia in mice

**Figure 8:**
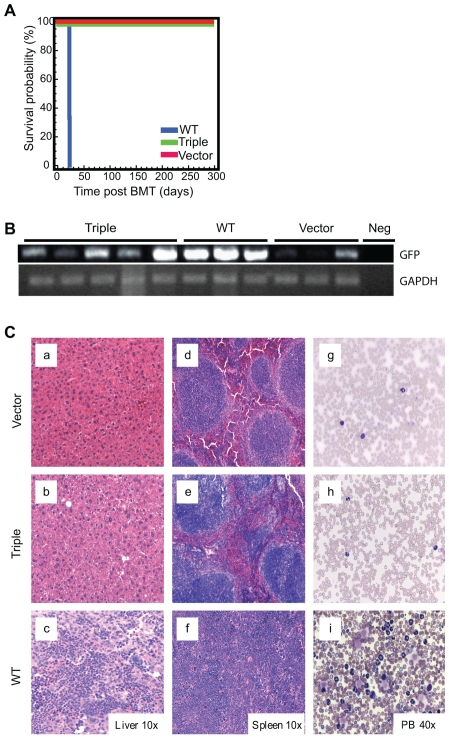
BCR-ABL triple mutant fails to induce leukemia in mice. Murine bone marrow transplantation studies were performed, transplanting bone marrow infected with retroviral supernatant from MIG-BCR-ABL-wild type, MIG- BCR-ABL-triple mutant or MIG vector alone, into lethally irradiated recipients. Mice were monitored post transplant for signs of disease onset. (A) Survival curves for the three recipient populations. (B) Green fluorescent protein (GFP, upper panel) or glyceraldehyde-3-phosphate dehydrogenase (GAPDH, lower panel) was amplified by RT-PCR from RNA purified from spleens of triple mutant, wild type or vector only recipients and visualized on agarose gels. (C) H&E stain from the liver (a–c), spleen (d–f) and Wright/Giemsa stain from peripheral blood smear (g–i) of representative mice at harvest; vector only (upper panels), triple mutant (center panels) and wild type (lower panels).

To assess the ability of the triple mutant to induce a myeloproliferative disease *in vivo*, lethally irradiated Balb/c mice were transplanted with bone marrow transduced with wild type, triple mutant or empty vector. Assays were carried out using retroviral supernatants of MIG-wild type, the MIG-triple mutant and MIG vector only, matched for GFP expression. As shown in Figure 8A, the triple mutant failed to induce leukemia. Four of 5 mice transplanted with bone marrow infected with the triple mutant vector survived up to 294 days post transplant when they were sacrificed for pathological examination of tissues. A fifth mouse was sacrificed after 118 days for pathological examination to look for evidence of disease. For comparison, mice transplanted with wild type virus had a post-transplant survival of between 20–24 days (Figure 8A). Peripheral blood counts from mice transplanted with the triple mutant and vector only were in the normal range (Table 1). The tissues harvested from the triple mutant mice showed no obvious morphological abnormalities (Figure 8C; b, e, h), and were similar to the vector only control animals (Figure 8C; a, d, g). In contrast the wild type mice exhibited massive expansion of leukemic cells in the liver, spleen and peripheral blood (Figure 8C; c, f, i). Southern blots were performed using genomic DNA from the spleens and bone marrow of the transplanted mice to analyze for integration of viral DNA, using the IRES from the MIG vector as a probe. Proviral integration was only seen in the wild type mice and in the bone marrow of one of the vector only mice (data not shown). As the sensitivity of Southern blot analysis is relatively low, PCR was performed to amplify GFP from RNA isolated from the spleen of each mouse, to ensure that there was expression of the retroviral constructs in each animal. As shown in Figure 8B, all the mice transplanted with the triple mutant expressed the retroviral vector, suggesting the presence of a small population of cells transduced with the retroviral construct was present, but failed to expand.

**Table 1:** Clinical characteristics of mice transplanted with MIG vector, MIG-WT and MIG-triple mutant.

BCR-ABL construct	Disease	WBC (x106/L)	Lym (x106/L)	Mon (x106/L)	Gra (x106/L)	Liver (g)	Spleen (g)
WT (n = 6)	CML-like	201±69	20.7±5.8	6.63±2.95	174±66	2.62±0.28	0.82±0.13
Triple (n = 5)	Normal	12.8±2.4	6.32±1.09	0.96±0.26	5.52±1.26	1.14±0.07	0.10±0.01
MIG vector (n = 6)	Normal	15±3.6	7.73±2.5	1.47±0.27	5.8±1.14	0.92±0.12	0.09±0.02

See text for details. Pre-moribund mice transplanted with MIG vector, MIG-wild type and MIG-triple were sacrificed for pathological evaluation. Counts of total white blood cells (WBC), lymphocytes (Lym), monocytes (Mon) and granulocytes (Gra) were analyzed from peripheral blood on a VetABC blood analyzer. Livers and spleens were harvested, weighed and analyzed histologically.

## Discussion

The BCR-ABL protein is known to associate with and activate numerous cellular signaling proteins. The emerging view is that BCR-ABL assembles a multi-protein complex whose signaling output leads to cellular transformation. Deciphering the contribution to transformation of individual domains of BCR-ABL or signaling proteins has been problematic as many proteins can interact both directly and indirectly with BCR-ABL. For example, deletion of a proline-rich motif in the C-terminus of BCR-ABL abolishes direct binding of BCR-ABL to the N-terminal SH3 domain of CRKL; however, CRKL remains tyrosine phosphorylated and associated with BCR-ABL in cells expressing this deletion mutant [24]. This is likely the result of interactions between CRKL and other adaptor or signaling molecules, such as c-CBL or p62^DOK^ which have been shown to link CRKL indirectly to BCR-ABL [22], [23]. In addition, an individual domain or binding site can mediate binding to more than one protein. For example, the SH2 domain mediates an association of BCR-ABL with CBL [21], [22] and p62^DOK^ [23]. Even the use of animals that lack specific signaling proteins has not been particularly revealing. In some cases, such as GRB2, the null phenotype is embryonic lethal [29]. In others, such as c-CBL null animals, no defect in BCR-ABL transformation has been observed [30]. This result could be interpreted as a lack of necessity of the specific protein for BCR-ABL function or compensation for the deleted gene by other proteins.

To investigate the necessity of various signaling proteins for BCR-ABL, we sought to identify a BCR-ABL mutant that remained kinase active, yet lacked transformation capacity. This was accomplished by the construction of a triple mutant including the Y177F substitution in BCR and deletions of the SH2 and the C-terminal proline-rich domains of ABL. In isolation the Y177F, ΔSH2 and ΔPro single mutants of BCR-ABL are capable of rendering myeloid cells IL-3 independent for growth, but they are much less potent than wild type BCR-ABL for transformation of fibroblasts. In a murine bone marrow transplantation model both the Y177F and ΔSH2 mutants induced leukemia *in vivo*, however in contrast to the myeloid phenotype of the wild type mice the phenotype of these single mutants was lymphoid and latency was increased [16], [20]. The C-terminal proline-rich domain has been examined in the p185^BCR-ABL^ background as a single mutation and in combination with deletion of the ABL SH3 domain. Mutants expressing a deletion of the proline-rich domain alone and in combination with the SH3 domain were also capable of transforming hematopoietic cells to factor independent growth. The single mutations induced a leukemia similar to wild type when transplanted into mice, however the double mutant induced an undetermined disease with recipients surviving twice as long as wild type recipient mice [25].

Using a triple mutant of BCR-ABL (Y177F, ΔSH2, and ΔPro), we show that this mutant still confers growth factor independence to 32D cells (Figure 1), but exhibits a profound defect in the transformation of primary hematopoietic cells. To this end, we show that in Whitlock-Witte B-cell transformation assays the ability of the triple mutant to induce outgrowth is completely abrogated. Furthermore, the ability to form myeloid colonies in the absence of cytokines is severely compromised relative to wild type (Figure 7). Consistent with these observations, the triple mutant was found to be incapable of inducing leukemia *in vivo* in a bone marrow transduction/transplantation model (Figure 8). Thus, the mutant described here represents the first report of a BCR-ABL mutant retaining kinase activity that is completely transformation deficient *in vivo*. *In vitro* kinase assays revealed that the kinase activity of the triple mutant, although reduced compared to wild type, is equal to the activity of the ΔSH2 single mutant (Figure 2). As the ΔSH2 mutant retains transformation competency in both cell lines and primary cells [17], [19], [20], this strongly suggests that the reduced kinase activity is not causing the transformation defect.

Examination of the canonical BCR-ABL signaling pathways in 32D cells showed that only STAT5 activation was maintained at wild type levels, suggesting that activation of STAT5 may be sufficient to induce IL-3 independence (Figure 5). This is consistent with a report by Onishi and colleagues, that a constitutively active STAT5 mutant is capable of inducing IL-3 independent growth [31]. In contrast, the significantly reduced activity of the MAP kinase and PI3-kinase/AKT pathways suggest that they are not required for BCR-ABL to induce growth factor independence in the triple mutant cell lines. This is consistent with the findings of Cortez et al, that RAS activation alone is not sufficient for transformation by BCR-ABL [28]. In addition to STAT5 we found that tyrosine phosphorylation of p62^DOK^ is maintained in the triple mutant (Figure 4), in contrast to CBL and CRKL tyrosine phosphorylation. Thus it is possible that p62^DOK^ phosphorylation in addition to STAT5 is required for factor-independent growth. Many groups have attempted to determine the role of phosphorylated p62^DOK^ in cellular signaling and suggest that phosphorylated p62^DOK^ plays a role in the negative regulation of growth factor stimulated pathways and/or RAS signaling [32]. Our data suggest that CBL and CRKL phosphorylation are primarily required for modulation of cellular functions other than factor independent proliferation and survival. For example, signaling through CRKL could affect adhesion and migration via interactions with paxillin, CAS and CBL and by forming complexes with focal adhesion kinases [33]. It has also been suggested that CRKL may play a role in signaling via its interactions with integrins, B- and T-cell receptors and various cytokines [34]. CRKL and CBL have been shown to interact in a multi-protein complex along with PI3-kinase when phosphorylated in BCR-ABL expressing cells [21]. Additionally, phosphorylated CRKL can transform fibroblasts through association with SOS and C3G, thereby activating the RAS pathway, analogous to the role of GRB2 in the activation of the same pathway [27]. The pattern of tyrosine phosphorylation in the BCR-ABL triple mutant points out the complexity of BCR-ABL signaling. We have shown that CRKL co-immunoprecipitates with the ΔPro mutant of BCR-ABL in cellular lysates, although this mutant is not capable of directly binding CRKL [24], and suggested that this interaction may be mediated by an association between CRKL and CBL, which in turn binds to the ABL SH2 domain in a tyrosine dependent manner. However, in the triple mutant, CRKL tyrosine phosphorylation and the association of CRKL and BCR-ABL are maintained, although the interaction between BCR-ABL and CBL is significantly reduced (Figure 3). As we know that there is no direct interaction between BCR-ABL and CRKL in the ΔPro mutant, this suggests that an unidentified protein(s) mediates the indirect interaction between CRKL and BCR-ABL.

Despite activation of STAT5, the BCR-ABL triple mutant is not able to induce leukemia *in vivo*. As noted, several signaling pathways are significantly impaired in this mutant, including the MAPK and PI3-kinase/AKT pathways (Figure 5). The fact that the respective single mutants are leukemogenic suggests that the combination of all three mutations abolishes leukemogenicity by overcoming the redundancy of the system. For example, the BCR-ABL Y177F mutant is deficient in GRB2 binding, but still activates RAS in 32D and BaF3 cells, likely via SH2 domain dependent interaction with GRB2 and SHC [15]. The fact that RAS activation via the SH2 domain is preserved in the Y177F mutant could explain why this mutant still maintains leukemogenicity *in vivo*. In addition, the SH2 domain of BCR-ABL has been shown to be required for activation of AKT through its association with the p85 subunit of PI3-kinase [35]. The combined impairment of PI3-kinase and RAS activation may explain the triple mutant's inability to transform primary hematopoietic progenitor cells.

It is not entirely clear which precise functional defect is responsible for the loss of leukemogenic potential. Bone marrow containing similar percentages of GFP positive cells (Table S1) of vector control, wild type or triple mutant were used for transplantation. Proviral integration in hematopoietic tissues from mice transplanted with bone marrow infected with the triple mutant was detected by PCR (Figure 8B), but not Southern blotting, indicating that only a small fraction of hematopoietic cells contained the triple mutant construct. Our results suggest that hematopoietic cells transduced with the triple mutant are at a selective disadvantage compared to cells transduced with wild type BCR-ABL or empty vector, as their proportion was reduced after engraftment. Further experiments will be required to determine whether this reflects failure of the cells to engraft or failure to expand after engraftment. The data from the B-cell and myeloid colony assays are clearly much more compatible with the second possibility. The data from the primary hematopoietic cells also support this assertion. Given the evidence demonstrating expression of BCR-ABL and of comparable levels of downstream signaling in primary cells (Figure 6), the identical nature of the vector backbone in all three constructs used and the fact that cell lines with high expression of the triple mutant were easily derived, low expression of the triple mutant does not appear to be a plausible explanation for the lack of disease in the mice transduced with the triple mutant bone marrow.

In summary, we show that mutation of BCR-ABL tyrosine 177 to phenylalanine in combination with deletion of the SH2 and proline rich domains abolishes the capacity of BCR-ABL to transform primary hematopoietic progenitor cells, but remains capable of inducing factor-independent growth in cell lines. This implies that a combination of small molecules that simultaneously block Y177 [36], the protein binding SH2 domain and the proline rich region could suppress BCR-ABL induced leukemia despite maintenance of kinase activity.

## Materials and Methods

### Construction of BCR-ABL^p210^ mutants

Constructs containing the point mutation of tyrosine 177 to phenylalanine (Y177F) [13], deletion of the ABL SH2 domain (ΔSH2) [17], and deletion of the proline-rich region of the C terminus (ΔPro) [24], have been described previously. A “triple mutant” construct containing all three changes was created from wild-type BCR-ABL by a series of standard subcloning steps in a modified pGEM5z vector (Promega, Madison, WI, USA). We recognize that since BCR-ABL is a fusion protein that is not naturally occurring, the term “wild type” is technically incorrect. However, cumbersome terminology such as “BCR-ABL with no mutations in the coding sequences of BCR and ABL” would be required to accurately describe the starting clone. For this reason, we have chosen to use the term wild type to describe the fusion protein that is observed in patients with CML. The wild type, Y177F, ΔSH2, ΔPro and triple mutant forms of BCR-ABL were excised from the modified pGEM5z vector as EcoR1 cassettes and subcloned into the pSRα-MSV-tk-neo retroviral expression vector (pSRα) [37], yielding plasmids pSRα-p210-wild type (WT), pSRα-p210-Y177F (Y177F), pSRα-p210-ΔSH2 (ΔSH2), pSRα-p210-ΔPro (ΔPro) and pSRα-triple mutant (triple) for expression in 32Dcl3 cells. Constructs were also subcloned into the MSCV-IRES-GFP (MIGR1) [2], yielding plasmids MIG-p210 wild type (WT) and MIG-triple mutant (triple) for use in B-cell lymphoid outgrowth assays, colony forming assays and murine bone marrow transplantation experiments.

### Cells and cell culture

The 32Dcl3 cell line (herein referred to as 32D cells) was obtained from Joel Greenberger, University of Massachusetts Medical Center, Worcester, MA [38]. 32D cell lines expressing wild type, Y177F, ΔSH2, ΔPro and the triple were generated by electroporation or by infection with retroviral supernatant generated from 293T cells [39]. Mass cultures were expanded in RPMI-1640 supplemented with 10% fetal bovine serum (FBS) and 15% WEHI-3B conditioned media as a source of murine interleukin-3 (IL-3). Individual clones were isolated by plating cells in 0.25% soft agar (Seakem, FMC Bioproducts, USA) in complete media containing 15% WEHI-3B conditioned media. Individual colonies were picked after 10–14 days and expanded in RPMI-1640 supplemented with 10% FBS and 15% WEHI-3B conditioned media. With IL-3 in the media, selection for growth factor independence was avoided. Individual clones were analyzed for BCR-ABL expression by immunoblot analysis. K562 cells, a BCR-ABL positive cell line [40], were cultured in RPMI-1640 supplemented with 10% FBS. Imatinib, kindly provided by E. Buchdunger (Novartis, Basel, Switzerland) was prepared freshly as a 10 mM stock solution in sterile phosphate-buffered saline (PBS) and diluted in RPMI 1640 medium immediately prior to use.

### Cell proliferation and viability assays

Factor independent growth was assessed with a tetrazolium compound [3-(4,5-dimethylthiazol-2-yl)-5-(3-carboxymethoxyphenyl)-2-(4-sulfophenyl)-2H-tetrazolium, inner salt (MTS) (Promega, Madison, WI, USA) assays as described [41]. Briefly, 1×10^4^ cells were plated in quadruplicate wells of four separate 96 well plates, with and without IL-3. MTS was added to one plate, which was incubated at 37°C for 4 hours, followed by measurement of the optical density (OD) at 490 nm. This was repeated at 24 hour intervals with the remaining three plates. For cell viability assays, 2×10^6^ cells were cultured in T25 flasks, with and without IL-3. Viable cells that excluded trypan blue were counted daily for 7 days.

### Kinase assays

Immunoprecipitated wild type, ΔSH2, and triple mutant BCR-ABL proteins from the 32D cell lines were used to measure the relative kinase activity of the proteins. Kinase activity was assayed using a synthetic NH2-terminal biotin linked peptide substrate containing the consensus binding sequence for the ABL kinase (biotin-EAIYAAPFAKKK-amide; New England Peptide, Gardner, MA, USA) [42], [43]. Briefly, 32D cell lysates normalized for BCR-ABL expression were immunoprecipitated using the ABL antibody (K12, Santa Cruz Biotechnologies, Santa Cruz, CA, USA) followed by incubation with protein-A-sepharose. Immunoprecipitates were washed extensively in kinase buffer (25 mM Tris-HCl, pH 7.5, 5 mM β-glycerophosphate, 2 mM DTT, 0.1 mM sodium orthovanadate [Na_3_VO_4_], 10 mM MgCl_2_) and resuspended in the same. Kinase assays were incubated at 30°C for 10 minutes in kinase buffer plus 50 uM ATP, [γ^32^P]ATP at 5000–5000 cpm/pmol and 2 uM peptide substrate. Assays were terminated with guanidine hydrochloride. A portion of the assay was spotted onto streptavidin coated membranes (SAM^2^®, Promega, Madison, WI), washed and dried as recommended by the manufacturer. Phosphate incorporation was detected by liquid scintillation counting. Assays were performed in triplicate for each BCR-ABL protein, with and without imatinib in the assay to control for kinase activity due to other kinases aside from BCR-ABL that might be present in the immunoprecipitates. Background binding was corrected using reactions containing no peptide. Counts were averaged in each assay, corrected for background counts and for BCR-ABL expression, as determined by Western blotting. Three replicate assays were performed and the activity relative to p210 wt was calculated.

### Immunoprecipitations and immunoblotting

Cells were lysed in NP40 lysis buffer (20 mM Tris, pH 8.0, 1 mM EDTA, 150 mM NaCl, 1% NP40, 10% glycerol, also containing 10 ug/mL aprotinin, 1 mM Na_3_VO_4_ and 1 mM phenylmethylsulfonyl fluoride). Equal amounts of whole cell lysate were run on SDS-PAGE gels and transferred to PVDF membranes (Immobilon-P, Millipore, Temecula, CA, USA) for 4 hours at 0.55 amps in 25 mM Tris, 192.5 mM glycine and 20% methanol. Non-specific binding sites were blocked with either 2.5% gelatin (for anti-phosphotyrosine blots) or 5% non-fat milk in TBS-T (10 mM Tris, pH 8.0, 150 mM NaCl, 0.05% Tween20) for 1 h at 25°C. The blots were incubated at room temperature with anti-phosphotyrosine antibodies (4G10), from Millipore, or one of the following antibodies from Cell Signaling (Beverly, MA, USA): phospho-AKT (S473), phospho-MAPK (T202/Y204), phospho-STAT5 (Y694) or phospho-MEK (S217/221). Blots were stripped and reprobed with antibodies recognizing AKT, MAPK, STAT5 (Santa Cruz Biotechnology, Santa Cruz, CA, USA) and MEK (Cell Signaling), respectively. Antibody reactions were detected by enhanced chemiluminescence (Pierce, Rockford, IL, USA) and quantitated using the Lumi Analyst software (Boehringer Mannheim, Roche Diagnostics, Indianapolis, IN, USA). For immunoprecipitation studies, equal amounts of lysate were incubated with antibody followed by incubation with either protein A- or G-sepharose. Antibodies used for immunoprecipitations were: c-CBL (C-15), c-ABL (K12), CRKL (C-20) and STAT5 (C-17) from Santa Cruz and p62^DOK^ from Covance Research Products (Grand Rapids, MI, USA). Immunoprecipitated lysates were separated on SDS-PAGE gels and transferred to PVDF membranes as above and the membranes were incubated with the following antibodies: anti-phosphotyrosine (4G10), anti-GRB2 (3F2), Millipore Corporation, anti-ABL (2411), anti-CRKL (C20), anti-STAT5 (C-17) and anti-p62^DOK^ (A3) from Santa Cruz and anti-CBL from BD Biosciences (San Diego, CA, USA).

### Generation of retrovirus

Bosc23 cells [39] were maintained in Dulbecco's Modified Eagles Medium (DMEM) supplemented with 10% FBS, 1 U/mL penicillin, and 1 µg/mL streptomycin. For production of retrovirus, Bosc23 cells were transiently transfected with MIG-WT, MIG-Triple or control MIG vector retrovirus, using Fugene (Roche). The viral supernatants were harvested after 48 hours post-transfection. For titration of retroviral supernatants, 100,000 NIH3T3 cells were infected in 35 mm plates with graded amounts of supernatant [44]. After 48 hours the cells were analyzed for GFP expression by FACS using a BD FACSAria™ (BD Biosciences). Volumes of supernatant containing equal amounts of infectious particles were used to infect primary hematopoietic cells.

### Analysis of phospho-STAT5 in primary cells

Bone marrow was harvested from non 5-fluorouracil treated Balb/c mice, erythrocytes were lysed with HN_4_Cl/KHCO_3_ solution and the cells were incubated overnight at 37°C in DMEM supplemented with 10% FBS, 10% WEHI conditioned media, 6 ng/mL murine IL-3, 10 ng/mL murine IL-6 and 50 ng/mL murine SCF (PeproTech, Inc., Rocky Hill, NJ). The following day fresh media, plus 1 mM HEPES, 2 ug/mL polybrene (American BioAnalytical, Natick, MA) and matched retroviral stock for the triple mutant, wild type or vector control was added, and cells were subjected to two rounds of transduction and co-sedimentation 24 hours apart, separated by incubation overnight at 37°C[45]. After the second overnight incubation, GFP positive cells were sorted and collected with a BD FACSAria™ (BD Biosciences, San Jose, CA) followed again by incubation overnight in DMEM supplemented with 10% FBS, IL-3, IL-6 and SCF. Cells were washed twice and starved for 4 hours in DMEM without serum or cytokines. After starvation 5×10^5^ cells were fixed with BD™ Cytofix buffer (10 minutes on ice), permeabilized with BD™ Phosflow Perm Buffer III (30 minutes on ice), washed with BD Pharmingen™ Stain Buffer+FBS and stained with the Alexa Fluor® 647 mouse anti-Stat5 (pY694) antibody (30 minutes at room temperature, in the dark), followed by analysis on a BD FACSAria™. Remaining cells were lysed for western analysis of pSTAT5 (Y694) (Cell Signaling) and BCR-ABL (c-ABL, Santa Cruz), stripped and re-probed for STAT5 (Santa Cruz).

### B-lymphoid transformation

To analyze the transformation of primary bone marrow B-lymphoid progenitors, bone marrow from non 5-fluorouracil treated mice was used [4]. Erythrocytes were lysed with HN_4_Cl/KHCO_3_solution and the cells were subjected to a single round of transduction and co-sedimentation with matched retroviral stock for the triple mutant, wild type and vector control in DMEM supplemented with 10% FBS. Cells were incubated overnight in the presence of viral supernatant. The cells were then plated for *in vitro* growth in Whitlock/Witte cultures in RPMI 1640 supplemented with 5% FBS, 200 µM L-glutamine, 50 µM 2-mercaptonoethanol and penicillin/streptomycin as described [20], [46]. Cells were plated in triplicate in serial dilutions at 1×10^5^, 3×10^4^, 1×10^4^, 3×10^3^ and 1×10^3^ cells/mL, along with 1×10^6^ non-transduced bone marrow cells. Cells were cultured for three weeks and fed twice weekly by the removal of 0.5 mL of supernatant and addition of 0.5 mL of fresh media. Cultures were scored as positive for transformation when the number of non-adherent cells exceeded 10^6^ per mL of culture medium.

### Myeloid Colony Forming Assays

For myeloid progenitor colony formation bone marrow was harvested from 6-10 week old female Balb/c mice. Bone marrow was subjected to 24 hours prestimulation at 37°C in IMDM (Invitrogen, Carlsbad, CA, USA) supplemented with 5% heat-inactivated FBS, 5% WEHI conditioned media, 6 ng/mL murine IL-3, 10 ng/mL murine IL-6 and 50 ng/mL murine SCF (Stem Cell Technologies, Vancouver, BC, Canada) [45]. After 24 hours of prestimulation, equal numbers of cells were transferred to 6-well plates and exposed to matched viral supernatants in the presence of 2 ug/mL polybrene in the same media as above. Cells were then co-sedimented at 30°C for 90 mins at 2500 rpm [44] and returned to the 37°C incubator overnight. After overnight incubation, cells were washed twice in IMDM to remove cytokines and plated in duplicate at 1×10^5^ cells in Methocult M3234 without cytokines or erythropoietin or in Methocult M3534 containing IL-3, IL-6 and SCF (Stem Cell Technologies). Cells were incubated at 37°C, 5% CO_2_ for 7 days after which the number of colonies were counted and are reported as a percentage of the number of wild type colonies formed. Genomic DNA was isolated from colonies using the Qiagen (Valencia, CA, USA) micro DNA kit and used in PCR as described below.

### Bone marrow transduction/transplantation

Murine bone marrow transduction and transplantation was performed as previously described [2]. Briefly, bone marrow cells were isolated from the tibias and femurs of 6-8 week old male Balb/c donor mice 4–5 days after intravenous treatment with 300 mg/kg of 5-fluorouracil (Sigma-Aldrich, St. Louis, MO, USA). Bone marrow cells (2×10^6^ cells) were infected with either MIG-WT, MIG-triple mutant-BCR-ABL or control MIG retroviral supernatant matched by titering in NIH-3T3 cells as described above, in DMEM (final concentration of viral supernatant of 35%), containing 1 U/mL penicillin, 1 µg/mL streptomycin, 2 mM L-glutamine, 15% FBS, 15% WEHI, 7 ng/mL interleukin-3, 12 ng/mL interleukin-6, 56 ng/mL stem cell factor, and 3 µg/mL polybrene, by two rounds of spinoculation [44]. Following infection, the cells were washed extensively in phosphate buffered saline (PBS) and 4×10^5^ cells were injected into the retro-orbital vein of recipient mice that had been exposed to two doses of 450 rad whole-body irradiation in a cesium irradiator administered 4 hours apart. After transplant, recipients were housed in microisolator cages supplied with water supplemented with antibiotics (Augmentin, OHSU pharmacy). Mice were monitored daily post transplant to look for signs of disease onset. White blood counts (WBC) and three-part differential blood counts were analyzed weekly using a Vet ABC blood analyzer (Heska Inc., Fort Collins, CO, USA).

### PCR amplification of GFP

Genomic DNA was isolated from myeloid colonies as described above. RNA was isolated from frozen tissue sections of spleens using the RNeasy isolation kit (Qiagen). cDNA was amplified using the Superscript III kit (Invitrogen) and used in PCR to amplify GFP from individual animals as described by Zhang, et al [2]. The GAPDH gene was amplified as a housekeeping control gene as described [47]Results were visualized on agarose gels.

### Southern Blot

Proviral integration was assessed by Southern blotting. Ten ug of genomic DNA from frozen spleen sections and bone marrow isolated at harvest was digested with EcoR1 and Sal1, run out on 0.8% agarose gel, transferred to Hybond-N+ (GE Healthcare, Piscataway, NJ, USA) and hybridized with a radioactive probe from the IRES gene in MIG-R1. Results were visualized on a Typhoon Imager (GE Healthcare).

### Pathological examination of diseased mice

For survival analysis, mice were monitored with weekly blood counts using a Vet ABC blood analyzer (Heska Inc.). Animals were sacrificed by CO_2_ asphyxiation when the white blood cell count exceeded 200/nl, if there was greater than 20% loss of body weight, or if they appeared moribund (IACUC guidelines, Oregon Health & Science University). For pathological analysis tissues were harvested, weighed and analyzed histologically, when the mice were sacrificed or when spontaneous death occurred. Paraffin-embedded thin sections of liver and spleen were stained with hematoxylin and eosin (H&E). Peripheral blood smears were stained with Wright/Giemsa stain. White blood counts (WBC) and three-part differential blood counts were analyzed from peripheral blood using the Vet ABC blood analyzer.

### Ethics Statement

All mice used in these experiments were housed and cared for in the OHSU Animal Care Facility under the supervision of the facility's veterinary staff. OHSU is an AAALAC accredited institution, meeting or exceeding all standards for animal care and use. This study has been reviewed and approved by the OHSU's Institutional Animal Care and Use Committee (IACUC). All procedures, such as injections and test bleeds, were performed by experienced personnel according to guidelines established by the IACUC, designed to ensure minimal discomfort and distress of the animals.

## Supporting Information

Figure S1 Growth curves of the triple mutant and BCR-ABL clones. Independent clones of 32D cells expressing either BCR-ABL wild type or triple mutant were isolated from soft agar cultures and their ability to grow in the absence of IL-3 was evaluated in cell proliferation assays. A representative cell proliferation assay with two wild type and four triple mutant clones is shown.
(0.30 MB TIF)

Table S1 Relative percentage of GFP positive cells in bone marrow used for transplantation. Flow cytometric analysis of the percentage of GFP positive bone marrow cells post infection with the indicated retroviral supernatants used in the bone marrow transplantation/transduction experiments.
(0.03 MB RTF)
