# Supplementary figures and images for: A BCR-ABL Mutant Lacking Direct Binding Sites for the GRB2, CBL and CRKL Adapter Proteins Fails to Induce Leukemia in Mice
Source: PLoS One. 2009 Oct 13;4(10):e7439. doi: 10.1371/journal.pone.0007439 (PMC2757918; doi:10.1371/journal.pone.0007439)

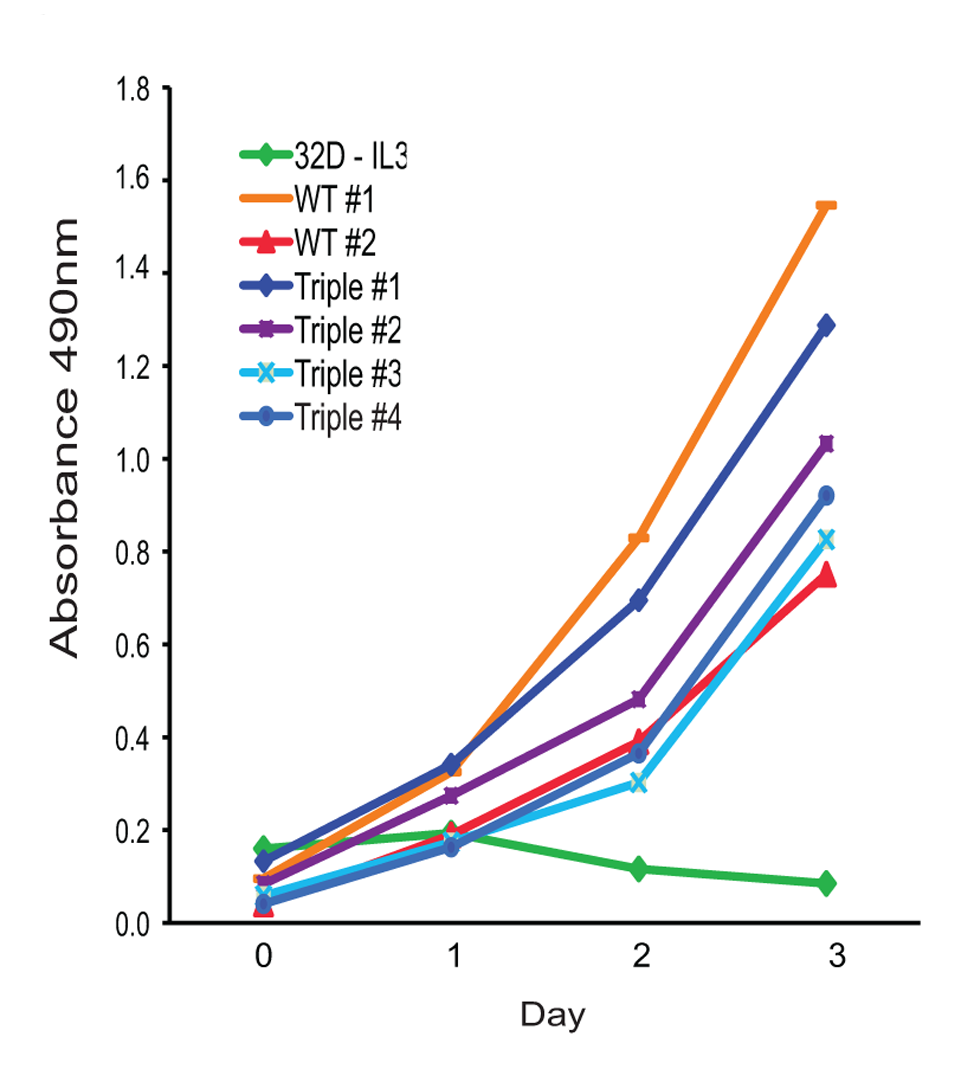

Supplement: Figure S1 — Growth curves of the triple mutant and BCR-ABL clones. Independent clones of 32D cells expressing either BCR-ABL wild type or triple mutant were isolated from soft agar cultures and their ability to grow in the absence of IL-3 was evaluated in cell proliferation assays. A representative cell proliferation assay with two wild type and four triple mutant clones is shown. (0.30 MB TIF) [file pone.0007439.s001.tif]
