# Supplementary material for: A BCR-ABL Mutant Lacking Direct Binding Sites for the GRB2, CBL and CRKL Adapter Proteins Fails to Induce Leukemia in Mice
Source: PLoS One. 2009 Oct 13;4(10):e7439. doi: 10.1371/journal.pone.0007439 (PMC2757918; doi:10.1371/journal.pone.0007439)
Supplement: Table S1 — Relative percentage of GFP positive cells in bone marrow used for transplantation. Flow cytometric analysis of the percentage of GFP positive bone marrow cells post infection with the indicated retroviral supernatants used in the bone marrow transplantation/transduction experiments. (0.03 MB RTF) [file pone.0007439.s002.rtf]

Table S1. Relative percentage of GFP positive cells in bone
marrow used for transplantation.
BCR-ABL construct	%GFP Positive Cells	
Wild Type	5.9	
Triple 	6.0	
MIG vector 	4.5	
